# Source location of volcanic earthquakes and subsurface characterization using fiber-optic cable and distributed acoustic sensing system

**DOI:** 10.1038/s41598-021-85621-8

**Published:** 2021-03-18

**Authors:** Takeshi Nishimura, Kentaro Emoto, Hisashi Nakahara, Satoshi Miura, Mare Yamamoto, Shunsuke Sugimura, Ayumu Ishikawa, Tsunehisa Kimura

**Affiliations:** 1 Department of Geophysics, Graduate School of Science, Tohoku University, 6-3 Aramaki-aza Aoba, Aoba-ku, Sendai, 980-8578 Japan; 2 Research Center for Prediction of Earthquakes and Volcanic Eruptions, Graduate School of Science, Tohoku University, 6-3 Aramaki-aza Aoba, Aoba-ku, Sendai, 980-8578 Japan; 3 Schlumberger SRPC, 1 Rue Henri Becquerel, BP.202, 92140 Clamart Cedex, France

**Keywords:** Natural hazards, Solid Earth sciences, Seismology, Volcanology

## Abstract

We present one of the first studies on source location determination for volcanic earthquakes and characterization of volcanic subsurfaces using data from a distributed acoustic sensing (DAS) system. Using the arrival time difference estimated from well-correlated waveforms and a dense spatial distribution of seismic amplitudes recorded along the fiber-optic cable, we determine the hypocenters of volcanic earthquakes recorded at Azuma volcano, Japan. The sources are located at a shallow depth beneath active volcanic areas with a range of approximately 1 km. Spatial distribution of the site amplification factors determined from coda waves of regional tectonic earthquakes are well correlated with old lava flow distributions and volcano topography. Since DAS observation can be performed remotely and buried fiber-optic cables are not damaged by volcanic ash or bombs during eruptions, this new observation system is suitable for monitoring of volcanoes without risk of system damage and for evaluating volcanic structures.

## Introduction

Volcanic earthquakes and tremors are generated by stress disturbances in a volcanic edifice caused by magma migration and/or pressurization of the magma chamber, conduit, cracks, or dikes. Their source locations have been used to clarify the magma system beneath active volcanoes. In addition, spatio-temporal changes of the source locations have been used to predict the locations of new eruptions and to monitor volcanic activity^1,2^. However, volcanic earthquakes and tremors that are generated by volcanic fluids (e.g., magma or hot water) are generally not well located because the onsets of P- and S-waves, which are used for standard hypocenter determination for tectonic earthquakes, are often unclear and/or seismic signals oscillate for a long time^3–5^.

In order to overcome such difficulties, small-aperture seismic arrays are used to measure tiny arrival time differences of seismic waves^6,7^. The seismometers in the array must be deployed close to each other, within a hundred meters, in order to maintain high coherency of seismic waves. Based on the propagation direction and apparent velocity of incident waves at more than two arrays, the hypocenter can be determined. However, the accuracy may not be high because wave propagation is greatly affected by heterogeneous structure beneath the arrays. Another way to overcome this difficulty is to use the spatial distribution of the seismic amplitude of volcanic earthquakes and tremors^8^. This method uses permanent seismic network data around a target volcano, and its simple analysis using the relation between amplitude attenuation and distance allows the method to be applied to volcanic earthquakes and tremors observed at many volcanoes^2,9^. However, the accuracy of the estimated source locations is affected by parameter assumptions. For instance, since volcanic subsurfaces consist of pyroclasts, old lava flows, steep valleys eroded by rainfalls and so on, evaluation of the site amplification factor and attenuation factor for seismic wave propagation affects the absolute location of the source^9^. Recently, seismic interferometry techniques have been developed and applied to volcanic tremor data^10–12^. In these techniques, cross-correlation functions (CCFs) for seismic waves recorded at several stations are stacked in order to retrieve direct waves propagating from the source location, and the time differences for the peaks of CCFs are used to determine the source location. Such methods, however, require continuous data lasting for several to tens of minutes in order to obtain stable cross-correlation functions for the source location.

Seismic observation using fiber-optic cable and a distributed acoustic sensing (DAS) system is now widely deployed in various geological fields for structure imaging, source studies, and hazard assessments^13^. The system can continuously record dynamic strain along the direction of the fiber-optic cable at an interval of 5–10 m for a distance of up to 40–50 km. For example, observations using a DAS system succeeded in recording regional earthquakes and teleseismic waves, and imaging active faults or seismic structures from the near-surface region to the Moho boundary^14–19^. Temporal seismic velocity changes in the fracture system have been investigated by applying seismic interferometry methods using ambient noise recorded by fiber-optic cables in boreholes^20^. The large number of channels recorded by DAS improves the sensitivity for detecting earthquakes below the noise level^21^.

The seismic observation network for active volcanoes generally consists of several to tens of seismometers deployed on the volcano flank and/or around active craters. Recently, some very dense seismic networks have been temporarily deployed at active volcanoes^22^, but temporal observations using a few tens to a hundred seismometers require a large amount of effort for the deployment of seismometers and frequent maintenance of the system. The present study focuses on the use of fiber-optic cable and a DAS system for monitoring volcanic earthquakes and tremors. Since the source location for volcanic earthquakes and tremors is one of the most important parameters for evaluating volcanic activity, we apply hypocenter determination methods that do not need onsets of P- and/or S-waves to the volcano seismic signals recorded by the DAS system. We apply two methods using (a) arrival time differences and (b) seismic amplitudes for volcanic low-frequency earthquakes, which are accompanied by tilt and/or very-long-period signals, observed at Mt. Azuma, Japan, and discuss the reliability and usefulness of the observations using DAS. We further estimate the site amplification factors of the subsurface structure by analyzing coda waves of regional tectonic earthquakes. We discuss the results with geological characteristics of the volcano, taking advantages of very high spatial resolution of fiber optic cable and DAS system.

## Field observations and data

**Figure 1:**
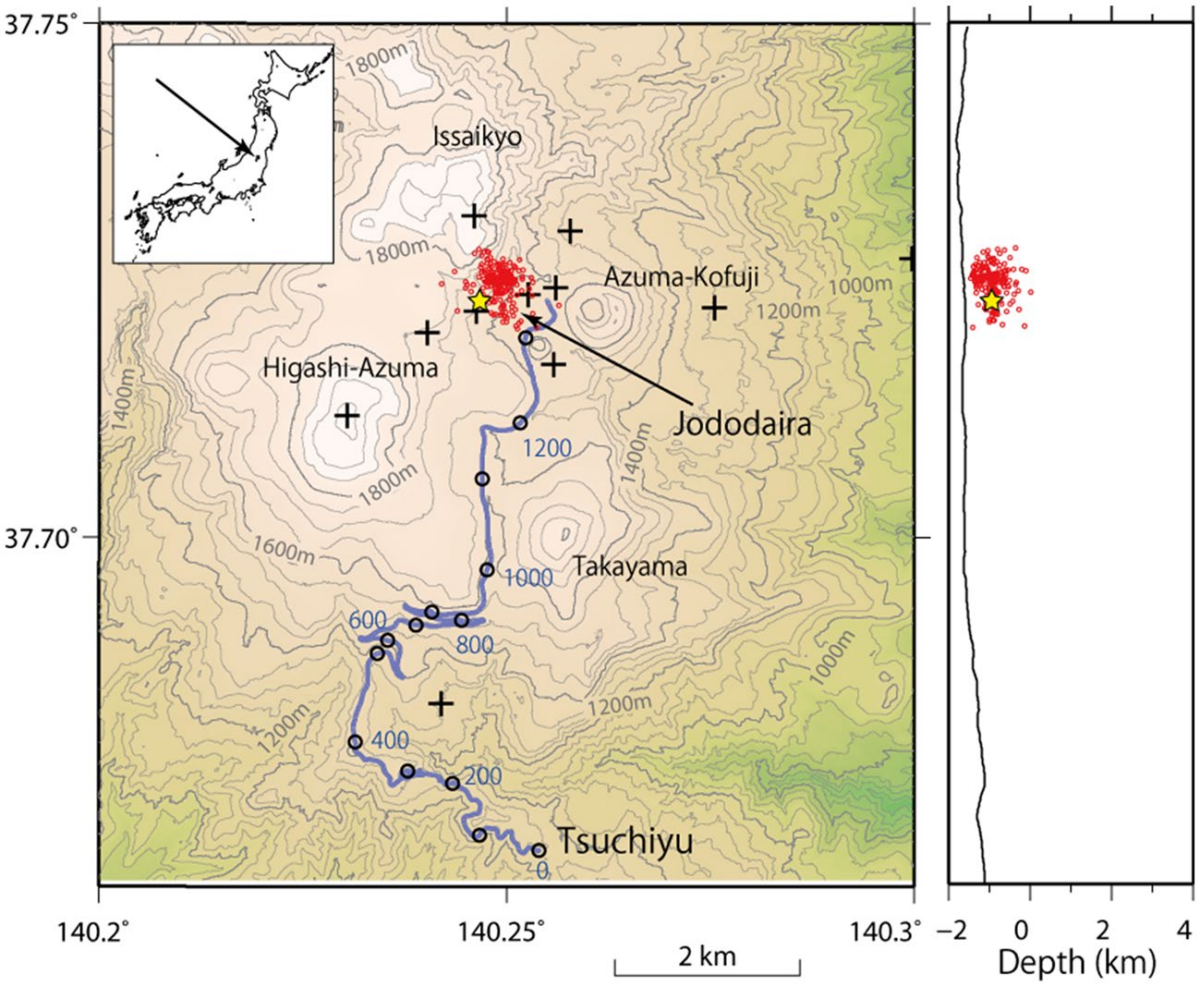
Locations of the fiber-optic cable and distributed acoustic sensing (DAS) system. The fiber-optic cable is indicated by the purple line, and the open black circles denote the locations of the measurement points every 200 number. Hypocenters of volcanic earthquakes determined by routine analyses of permanent station data for the period from January 1 to July 4, 2019 are indicated by red circles. Note that only the hypocenter of a volcanic earthquake on July 4, which is analyzed in the present study, is located by the routine analyses during our observation period. Plus symbols indicate permanent stations maintained by Tohoku University and the Japan Meteorological Agency. Azuma volcano consists of andesitic edifices, such as Issaikyo, Azuma-Kofuji, Higashi-Azuma, and Takayama. Volcanic activity during the Holocene period occurred around Jododaira. The DAS system is located at Tsuchiyu station, at the south end of the fiber-optic cable. Blue fine lines represent rivers. This figure was created by Generic Mapping Tools (GMT) v4.5.5^39^.

Figure 1 shows the locations of Azuma volcano and the fiber-optic cable we used in the present study. Permanent stations have been deployed by Tohoku University and the Japan Meteorological Agency (JMA), and short-period or broadband seismometers are installed at each station. The fiber-optic cable was deployed along a mountain road for a distance of 14 km. The cable was placed in corrugated hard polyethene pipes (FEP) embedded at a depth of approximately 50 cm. We performed observations for approximately three weeks from July 4 to July 25, 2019, installing a DAS system (heterodyne Distributed Vibration Sensing system (hDVS), Schlumberger) at Tsuchiyu station, at the southern end of the single-mode cable. We continuously recorded the dynamic strain signals in the direction along the cable with a sampling frequency of 1000 Hz, a spatial interval of 10.2 m, and a gauge length of 40.8 m. The data were resampled at 200 Hz in the following analyses. The DAS system uses a high-pass filter in order to eliminate nonlinear effects mainly due to temperature changes, and has a lowest frequency of 0.1 Hz (Methods, Observation using Fiber-optic cable and the DAS system).

## Source location determination for volcanic earthquakes

Azuma volcano is an active volcano located at the volcanic front of northeast Japan, and its eastern part consists of several andesitic edifices, such as Nishi-Azuma, Higashi-Azuma, Issaikyo, Takayama, and Azuma-Kofuj. Phreatic eruptions occurred around Issaikyo in 1893–1895, 1950, and 1977^23^. Fumarolic activity is still high around Jododaira (altitude: 1500 m), and intermittent and continuous seismic activity and inflation or deflation have been observed around Jododaira^24^. In Fig. 1, the hypocenters of volcanic earthquakes with relatively clear onsets of P -and S-waves recorded at the permanent stations are shown (Methods, Routine hypocenter determination using permanent station data). The hypocenters are distributed above sea level beneath Jododaira. Seismic activity is often associated with inflation-deflation activity detected by a borehole tilt meter, and is inferred to be caused by shallow hydrothermal activity^25^.

We examine six volcanic earthquakes that occurred during our observation period (Figure S1). The observed waveforms continued for approximately 10 s with a dominant frequency of 4 Hz. A volcanic earthquake that occurred at 12h35m (UT) on July 4, 2019, which was the largest volcanic earthquake during our observation period, was located 0.5 km beneath the ground surface at Jododaira based on routine hypocenter determination using the onsets of P- and S-waves. Its magnitude is estimated to be about − 0.1 from the maximum amplitude of ground velocity^26^. The other five volcanic earthquakes are not located enough accurately because of obscure P- and S-waves. Their magnitudes are estimated to be about − 1 if their hypocenters are located at the shallow depth beneath Jododaira. Note that all the hypocenters shown in Fig. 1 are the events occurring before the DAS observation except the volcanic earthquake on July 4.

Two methods are used for determining the source locations. One is the arrival time difference method, and the other method is the amplitude source location (ASL) method. The former method can determine source locations because the fiber-optic cable is deployed along a winding road with several curves that act as an L-shaped array, which enables us to infer the slowness (propagation direction and incident angle) of incident seismic waves. Its spatial resolution becomes worse for locations far from the cable. On the other hand, the spatial resolution of the ASL method becomes worse in the east–west direction because the cable mainly runs in the north–south direction (Methods, Source location errors). Hence, we determine the source location using both methods, and identify the most likely result. We used the waveform data with a signal to noise ratio of larger than 4 in the following analyses.

### Arrival time difference method

Volcanic earthquakes and tremor sometimes do not accompany any significant phases, such as P- or S-waves, but closely spaced measurement points along the fiber-optic cable allow measurements of very small arrival time differences for well-correlated waveforms between nearby measurement points. The arrival time differences change according to the incident angle of seismic waves to the measurement points along the cable. For example, the arrival time differences come to be small when the source is deep or when the source is located to the direction perpendicular to the direction of fiber optic cable. We use this principle to locate the sources of volcanic earthquakes.

**Figure 2:**
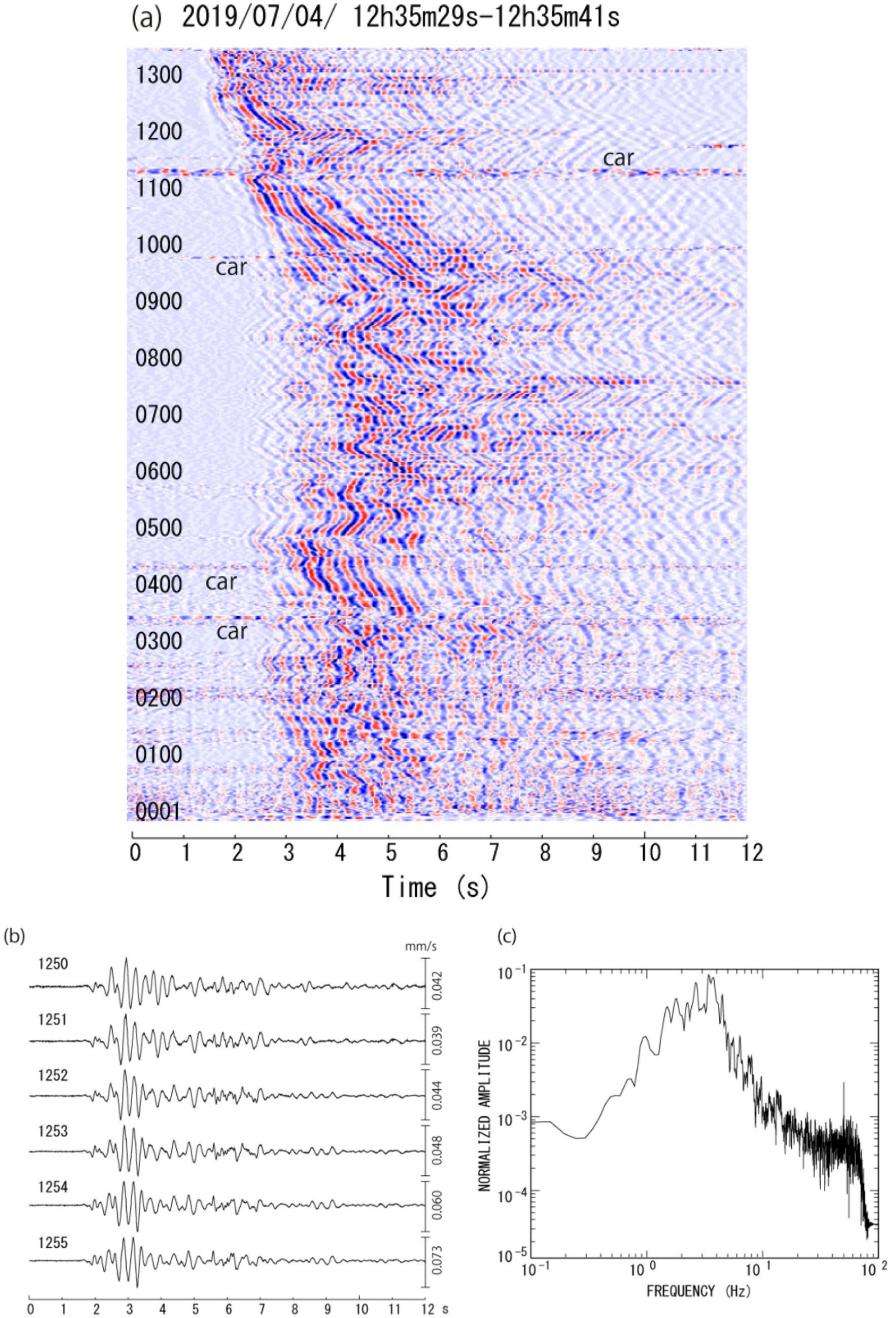
(**a**) Record section for the volcanic earthquake on July 4, 2019. (**b**) An example of a seismogram of the volcanic earthquake recorded at nearby measurement points. The amplitudes are converted by comparing the records of a short-period seismometer that was set closely at these measurement points. (**c**) Amplitude spectrum of the volcanic earthquake. Large amplitude signals that continue from the beginning to the end at several measurement points shown in (**a**) are noise generated by moving cars. The numbers shown at the left in (**a**) and (**b**) represent the measurement points.

We measure the arrival time differences for earthquakes recorded at two measurement points. Since the fiber-optic cable is deployed over a long distance, there are many choices for the distance between the two measurement points. In this analysis, distances of 30.6–71.4 m are used, for which highly correlated seismic waves are observed (Methods, Measurement of arrival time difference). The arrival time difference is calculated from the phase difference in the cross-spectra, applying a 12-s time window for the waveforms (Fig. 2). Since the cross-spectra are mainly calculated from waves with large amplitudes, we assume that the arrival time differences are associated with S-wave arrivals. The S-wave velocity structure used for routine hypocenter determination is assumed (Figure S2) and the station height is corrected for the calculation of theoretical travel times. We identify the best-fit location where the residual between the observed and theoretical arrival time differences is a minimum using a grid search method. The grid points are set every 0.02° in latitude and longitude and at depths of − 1.25, − 1, − 0.75, − 0.5, − 0.25, 0, 1, 2, and 3 km below sea level.

**Figure 3:**
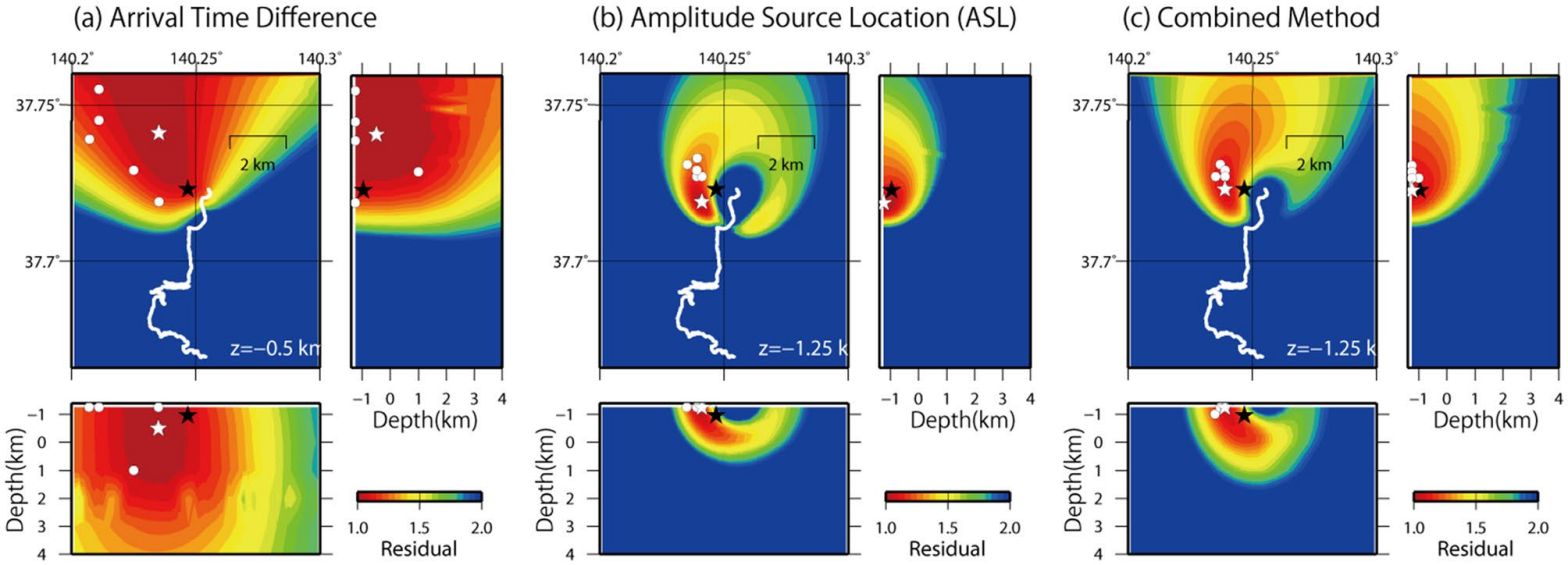
Source locations of the six volcanic earthquakes and spatial distribution of the residual between the observed and theoretical values for a volcanic earthquake at 12h35m on July 4, 2019. (**a**) Arrival time differences, (**b**) seismic amplitudes, and (**c**) combined method. White stars represent the estimated source locations of the earthquake on July 4 and white circles do those for the other five earthquakes. Black stars indicate the hypocenter obtained by routine hypocenter determination. White lines indicate the location of the fiber-optic cable. The residuals in (**a**) and (**b**) are normalized by the minimum residual for each method. The residual in (**c**) is S in Eq. (2). This figure was created by Generic Mapping Tools (GMT) v4.5.5^39^.

The source locations of the six volcanic earthquakes are widely distributed at depths of less than 1 km beneath the north-west region of Jododaira (Fig. 3a). Such horizontal extension of the source locations is caused by the fiber optic cable deployed mostly in the north–south direction in the southern area of Jododaira, which makes the small residuals run roughly in the north-west direction. Depth resolution may not be good as the small residuals extend down to 3–4 km depths. Source locations and residual maps as well as the measurement points used for the source locations are shown in Figure S3 for each of the six volcanic earthquakes.

### Amplitude source location method

We measure the maximum amplitudes of seismic waves along the fiber-optic cable in order to determine the source locations for the volcanic earthquakes. In the ASL method, the seismic wave generated from a source is assumed to be attenuated by geometrical spreading and intrinsic attenuation^8^. This method also supposes isotropic radiation of the seismic waves from a source. The source mechanisms are unknown for the volcanic earthquakes we analyzed, and even low-frequency volcanic earthquakes that are often expressed by a volumetric source such as a tensile crack^27^ may not isotropically radiate seismic waves. But, observed seismic waves may be approximated to be isotropically radiated from a source location^28^ because recent analyses of heterogeneous structures at active volcanoes indicate a highly heterogeneous shallow subsurface showing scattering mean free paths of approximately 1 km^29,30^. Hence, we represent the seismic amplitude $A$ at a distance $r$ from the source as

1 $$ \overline{A}_{i} = \frac{{A_{i} }}{{C_{i} }} = \frac{{A_{0} }}{{r_{i} }}\exp \left( { - \frac{{\omega r_{i} }}{2\beta Q}} \right), $$

where $Q$ is the quality factor for attenuation, $\omega$ is the angular frequency, $\beta$ is the S-wave velocity, $A_{0}$ is the seismic amplitude at the source, $C_{i}$ is the site amplification factor, and the subscript *i* indicates the measurement point. Site amplification factors are determined from analyses of coda waves for regional tectonic earthquakes (Methods, Estimation of site amplification factor).

We read the maximum amplitudes for the volcanic earthquake at all measurement points along the fiber-optic cable and correct them using $C_{i}$ to obtain $\overline{A}_{i}$. It is necessary to refine the amplitude by taking into account the difference between the fiber-cable direction and the seismic wave oscillation direction. However, the observed waves are inferred to be strongly scattered and therefore incident from many directions, as discussed in Discussion. Hence, such a correction is not applied in the following analyses. We assume an S-wave velocity $\beta$ of 3 km/s based on the velocity structure used in the routine hypocenter determination. We examine Q of 10–100 and the results do not change so much. In the following analyses, we use a $Q$ value of 20 that well explains the observation results. The residuals between $\overline{A}_{i}$ and the theoretical values are calculated for all grid points (Fig. 3b). The grid point with the smallest residual is determined as the best-fit location.

The source locations are determined at a very shallow depth of − 1.25 km beneath Jododaira. The source locations extend in the north–south direction, but seems to be less scattered than those determined by the arrival time difference method (Fig. 3b). Contrary to the residual distribution obtained using the arrival time difference method, a small residual region extends both to the east and west sides of the fiber-optic cable. The source depth appears to be well resolved as the residual becomes lager with depth, because the cable is deployed close to the source location (Figure S3).

### Combined method

In order to improve the spatial resolutions of the source location determined by the two methods, which is determined by the configuration of the fiber-optic cable and the data quality, we combine the two results based on the least squares method. We normalize the residuals separately using the minimum residual for each method and calculate the total residual at each grid point:

2 $$ S = \frac{1}{2}\left( {\mathop \sum \limits_{i}^{{N_{t} }} \frac{{(O_{t, i} - C_{t, i} )^{2} }}{{\sigma_{t}^{2} }} + \mathop \sum \limits_{i}^{{N_{a} }} \frac{{(O_{a, i} - C_{a, i} )^{2} }}{{\sigma_{a}^{2} }}} \right) $$

where $$O$$ and $$C$$ indicate the observed and theoretical values, respectively, $$\sigma^{2}$$ is the minimum residual (variance), $$N$$ is the total number of data points, and the subscripts $$t $$ and $$a$$ indicate the arrival time difference and amplitude, respectively. Figure 3c shows the spatial distribution of the source locations of the six volcanic earthquakes determined by the combined method. They are distributed at very shallow depth (− 1.25 km) beneath Jododarira. The horizontal extension is about 1 km and 0.5 km in the north–south and east–west directions, respectively. The extension is seen to be smaller than those independently determined from arrival time differences and amplitudes.

**Figure 4:**
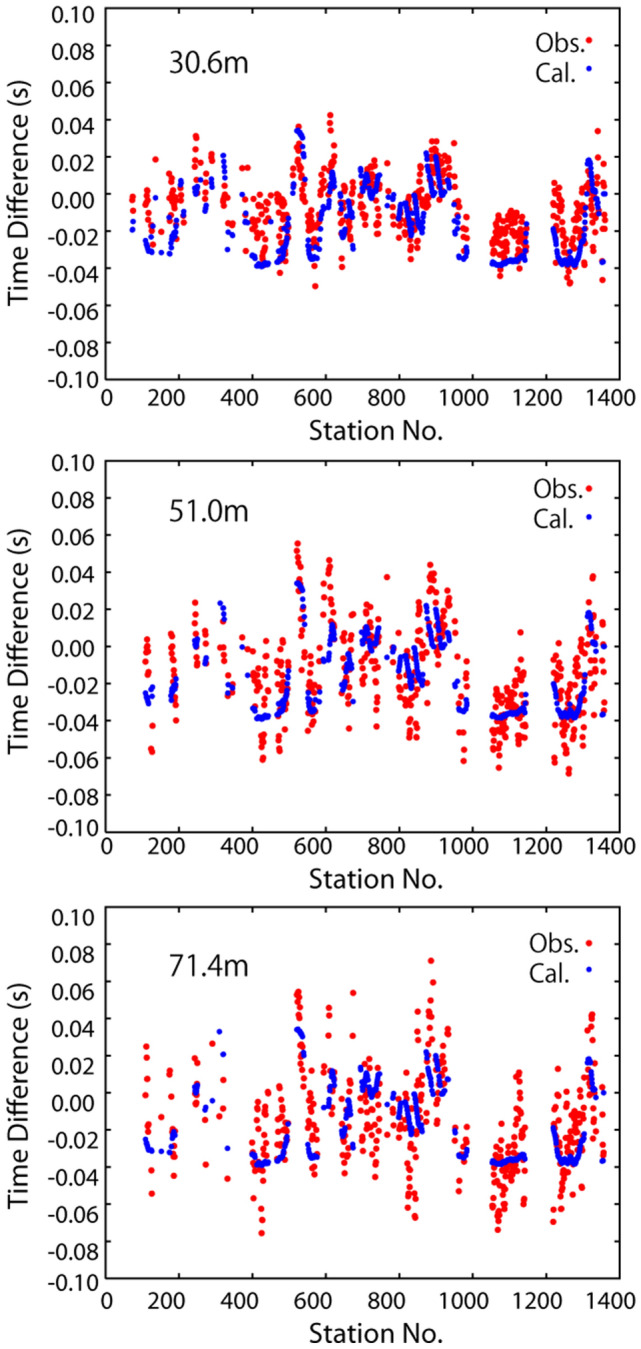
Comparison of the observed and theoretical arrival time differences for the best location for the July 4 earthquake. The measurement point differences are 30.6 m (left), 51 m (middle), and 71.4 m (right). Large station numbers represent locations close to Jododaira.

**Figure 5:**
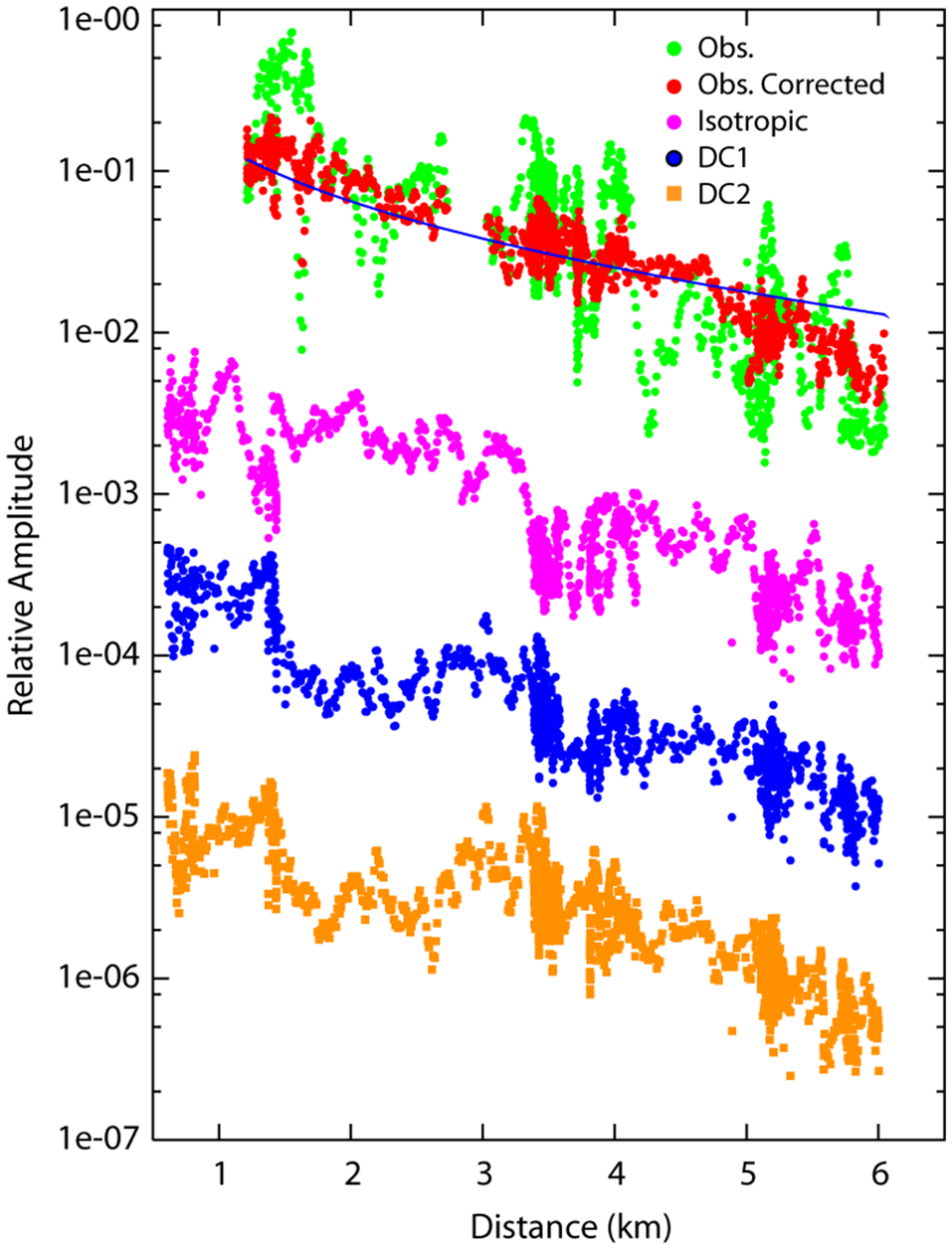
Comparison of the observed and theoretical amplitudes predicted for the best location for the July 4 earthquake. Green and red circles indicate the observed amplitude of dynamic strains and site factor corrected amplitudes, respectively. The black line indicates the theoretical prediction for the best location. Purple, blue, and orange symbols indicate theoretical amplitudes calculated by numerical simulations using an isotropic mechanism and two kinds of double-couple source mechanism. The theoretical amplitudes are shifted so as not to overlap each other.

Figure 4 compares the observed and theoretical arrival time differences calculated for the best source location for the July 4 earthquake. The observed data are well reproduced by the theoretical data, although the observed data are scattered. Figure 5 compares the observed and theoretical seismic amplitudes for the July 4 earthquake. The observed amplitudes corrected by site amplification factors, which gradually decrease with distance from the epicenters for the best source location, are well explained by the theoretical amplitudes. Note that the site amplification correction greatly reduces the fluctuations in the seismic amplitudes, which enable us to reliably determine the source location.

The hypocenters by the combined method are much affected by the ASL but less by the arrival time difference method for the six earthquakes we analyzed. This may suggest the usefulness of ASL, but the hypocenters by the combined method are concentrated in a smaller region. As shown in Methods (Source location errors), the spatial resolutions of the two methods depend on the source locations. Also, at other volcanoes, we need to use a fiber optic cable with different configuration. Hence, in the present study, we discuss the hypocenters determined by the combined method in the followings.

The source locations can be determined almost in real time using a standard PC with a Unix system by calculating the arrival time differences for all grid points beforehand.

## Subsurface structure obtained from analyses of regional tectonic earthquakes

**Figure 6:**
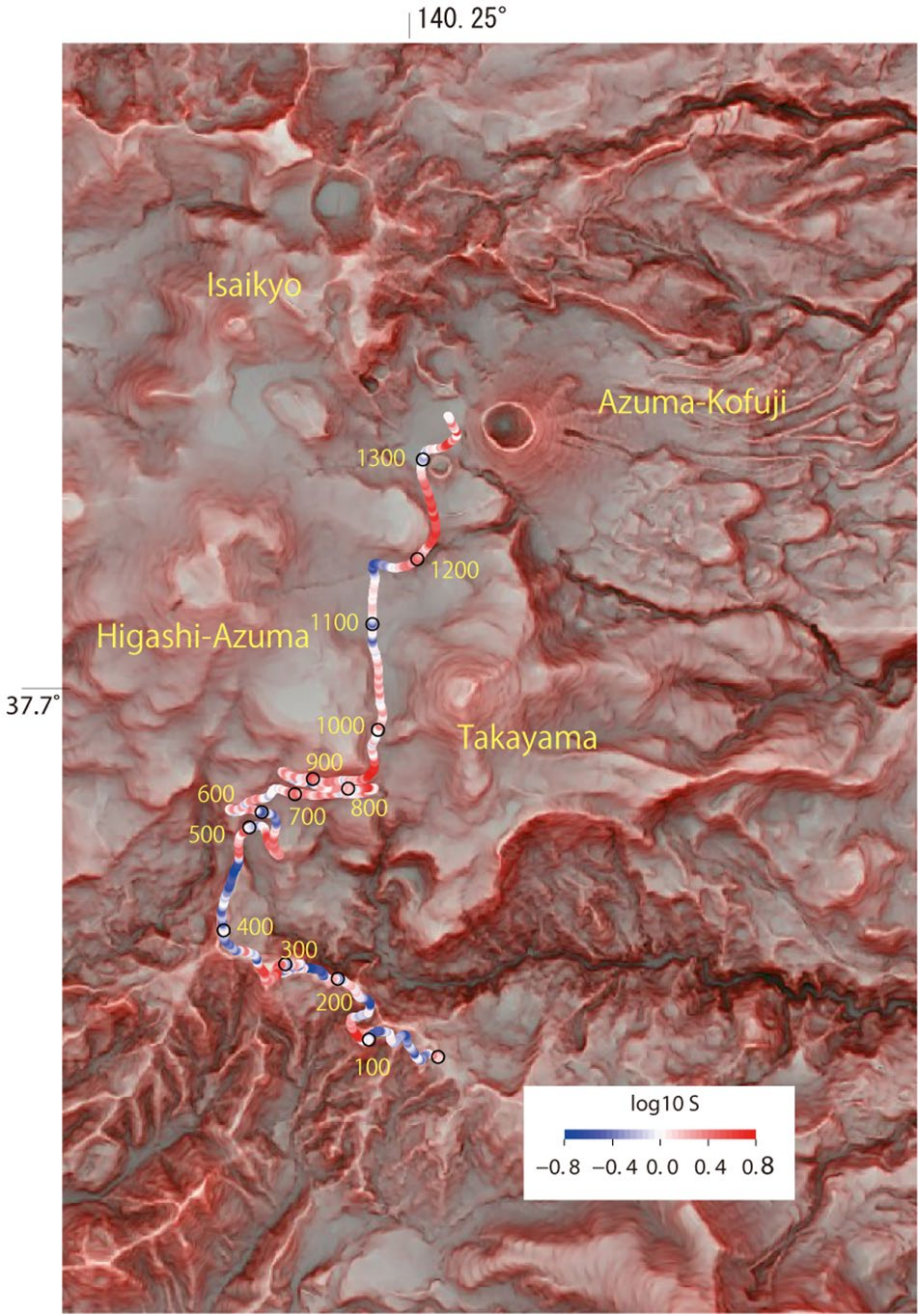
Site amplification factors along the fiber-optic cable. Logarithmic amplitudes of the factors are indicated by color contours. The black circles and corresponding numbers along the cable indicate the measurement point numbers. Back ground is a red relief map^31^ emphasizing topography of the volcano provided by the Geospatial Information Authority of Japan, which shows robes of old lava flows extending from the active volcanoes and steep valleys in the southern part.

The heterogeneity of the shallow structure of a volcano may also be clarified from the fiber-optic cable and DAS observations, as has been reported for different volcanic fields^16^. Figure 6 shows the site amplification factors estimated from coda wave analyses of regional tectonic earthquakes, which are used to correct the seismic amplitudes of the six volcanic earthquakes in Amplitude source location method. Small and large site amplification factors represent hard and soft subsurfaces, respectively. The site amplification factors are plotted on a red relief map emphasizing the topographic characteristics^31^. The site amplification factors are large in the northern parts of the cable for measurement point numbers (MPNs) larger than 500. This area is close to several active volcanoes at Azuma-Kofuji, Higashi-Azuma, and Takayama, and is characterized by a gentle slope consisting of pyroclasts and/or old lava flows. Closely looking at the map, we find large site amplification factors are located around the fronts of old lava flows that are recognized as robes extending from Higashi-Azuma (see the regions around MPNs 500–1100, MPNs 1180–1280). Large site amplification factors around MPN1330 are matched with the locations where sediments are accumulated. On the other hand, the southern area for MPNs smaller than approximately 500, which is far from these volcanoes, shows low site amplification factors. Since the region is located close to a valley and river (Fig. 6), small site factors may be related to erosion of the subsurface deposits by rainfall. Similar characteristics are also seen in the northern part, and a measurement point with an MPN of around 1150 showing relatively small site factors is located close to a small valley between Azuma-Kofuji and Takayama (see Fig. 1).

The volcano topography may influence the site amplification factors, because the shapes of the mountain and valley introduce focusing and/or defocusing phenomena of seismic wave propagation. We conducted numerical simulation of seismic wave propagation at the frequency band of 2–6 Hz, in which S-wave is vertically incident to the volcano, and measured root mean square amplitudes of the coda waves along the cable (Methods, Numerical simulation of seismic wave propagation). The results show that the simulated site amplification factors do not reproduce the amplitudes and spatial variations of the observed site amplification factors (Figure S5). This implies that the observed site amplification factors represent the characteristics of the subsurface structure of the volcano.

## Discussion

The volcanic earthquake on July 4 was routinely determined from analyses of the onsets of P- and S-waves at the permanent stations (Fig. 1), although the others are not because of unclear P- and S waves. The hypocenter of July 4 is well matched with the location where the minimum residual is obtained by the combined method (Fig. 3). The mismatch is approximately 500 m in horizontal direction and 250 m in depth. Such spatial resolution may enable us to relate the source locations with volcanic activity at the ground surface (e.g., eruption locations, main craters, and fumarolic area). The horizontal extension of the six earthquakes is about 1 km in horizontal directions. This is almost comparable to that of the hypocenters determined from P- and S-wave onsets, suggesting the usefulness of hypocenter determination using fiber optic cable and DAS. If the fiber optic cable was set surrounding the hypocentral regions, the misfits between the source locations determined by our method and those by the routine hypocenter determination come to be smaller as suggested from the simulation (Methods, Source location errors; Figure S6). Also, the use of a 3D seismic velocity model may reduce the misfits. Although the present study only examined six volcanic earthquakes because there was no volcanic tremor during the observation period, the method can be used to determine the source locations of volcanic tremors that show obscure P- and S-waves or continuous oscillations.

Fiber-optic cables can be buried so that we may continuously record the ground motion, even when pyroclasts and volcanic ash fall on the flank of volcanoes during explosive eruptions, which often destroys conventional permanent stations that use radio or Wi-Fi to transfer the signals to the data center. Also, malfunctions due to thunder, which often occurs in mountain areas, are expected to be greatly reduced in comparison with an observation system using electric cables and moving-coil-type seismometers. These advantages, as well as the ability to determine the source locations and site amplification factors, indicate the usefulness of observations using fiber-optic cable and the DAS system for monitoring volcanic seismicity and understanding volcano structures.

Particle orbits of the ground motion at 4 Hz, which are retrieved from three-component short-period or broadband seismometers, show complicated spheroidal shapes at the permanent stations located at a distance of greater than approximately 1 km from the hypocenter (Figure S4). This implies that seismic waves scattered by the heterogeneous structure arrive at the seismic stations from many directions. Since the measurement points along the fiber-optic cable are located at distances far greater than approximately 1 km, the observed dynamic strain signals consist primarily of scattered waves that reduce the effects of the direction of the fiber-optic cable. This enables us to apply the ASL method for the data recorded by DAS without correction for the direction of the fiber-optic cable.

In Fig. 5, we show several cases of seismic wave amplitudes calculated from numerical simulations in which the volcano topography is taken into account (Methods, Numerical simulation of seismic wave propagation). Three types of source mechanism are assumed at the hypocenter of the volcanic earthquakes. All of the simulation results show that the seismic amplitudes have local fluctuations, especially at epicentral distances of approximately 3.5–4 km and 5.2 km, and that they decay with distance. These local fluctuation characteristics are mainly observed at measurement points in regions with steep flanks, suggesting the effects of topography and/or the directions of fiber-optic cable. We also find that the theoretical seismic amplitudes at an epicentral distance of less than approximately 3 km differ for the three source mechanisms. The permanent stations located very close to the hypocenter (TU.JDD and V.AZHH in Figure S4) appear to show rectilinear particle orbits compared with the other stations at longer hypocenter distances, which suggests weak effects of the scattered waves and/or dominance of the near-field term. These results suggest the possibility of source mechanism estimation by analyzing data from nearby measurement points. If the fiber-optic cable is deployed close to the source regions and/or long-period waves are used^32^, it may be possible to apply the moment tensor inversion technique to determine the source mechanism for volcanic earthquakes, as has been done for the permanent station data. Stacking the waveforms along the fiber optic cable by taking into account the radiation pattern of source mechanism and the direction of fiber optic cable may also enable us to determine the source location.

Finally, we describe several applicability of fiber-optic observation with the DAS system to the evaluation of volcanic phenomena and other geological features. Calculating the relative amplitudes of coda waves of regional earthquakes can be used to evaluate the subsurface structure along the fiber optic cable in a fine scale not only at active volcanoes but also various geological settings such as active faults, sedimental layers and eroded rock sites. Estimated site amplification factors can be also used to quantitatively evaluate strong ground motions excited by large earthquakes. The method and analyses used in the present study may be applied to non-volcanic earthquakes and tremors occurring at plate boundaries, most of which also show emergent onsets of P- and S-waves^33,34^. Also, various kinds of geological phenomena such as debris flow after heavy rains and land slide are spatio-temporally located as migrations of the seismic sources. Development of broadband seismic observations using the DAS system, from a short period (1 s) to a very long period (10 s) as well as ground deformation can be useful in order to understand volcanic earthquakes and tremor, and various kinds of geological phenomena.

## Methods

### Observation using fiber-optic cable and the DAS system

We placed a heterodyne Distributed Vibration Sensing system (hDVS) by Schlumberger^35^ at the southern end of the fiber-optic cable (single mode), at Tsuchiyu station. The fiber-optic cable is maintained by the Ministry of Land, Infrastructure, Transport and Tourism (MLIT) to transfer various kinds of data that are used to monitor factors such as the road condition, meteorological information, and the activity of the crater. The fiber-optic cable is placed in corrugated hard polyethene pipes (FEP) embedded at a depth of approximately 50 cm. We continuously recorded dynamic strain in the direction along the cable with a sampling frequency of 1000 Hz, a spatial interval of 10.2 m, and a gage length of 40.8 m. We selected these parameters to measure phase differences for the dominant waves of volcanic earthquakes and tremors with a frequency of a few hertz and a wavelength of a few hundred meters. We performed a tap test to confirm the measurement point number at the location at which we artificially excited ground motion by hitting the ground surface. A total of 1383 channels were saved on hard disks every 30 s in SEGY format. The output of the hDVS system is the phase difference, which is proportional to the dynamic strain along the fiber. The strain signals (e.g., in Fig. 2; Figure S1 and S9) are converted to ground velocity^16,18^ by comparing the strain signal amplitude with the ground velocity measured by a short-period (0.5 s) seismometer that was installed at the site close to MNPs 0445, 1027, and 1268.

### Routine hypocenter determination using permanent station data

Volcanic earthquakes at Azuma volcano show various waveforms with dominant frequencies of up to about 10 Hz, sometimes accompanied by longer-period waves. Most of the waveforms show emergent onsets of P-waves and/or unclear S-waves, but some are accompanied by clear P- and/or S-waves. We routinely determine the hypocenters of volcanic earthquakes using P-wave and S-wave onsets. The hypocenters shown in Fig. 1 are those determined by using at least seven P- and S-wave onsets at more than five permanent stations, and their hypocenter location errors are about 150 m. The velocity structure shown in Figure S2 was used for hypocenter determination based on the P- and S-wave arrival times.

### Source location errors

The spatial distribution of seismic networks constrains the spatial resolution of hypocenters. Ideally, surrounding the hypocenter improves the horizontal accuracy, and stations just above the hypocenter constrain the depth. To observe active volcanoes, the fiber-optic cable should be placed above and surrounding the expected hypocenter location, but it may be necessary to use existing fiber-optic cables deployed along roads, which are not ideal for seismic observation. Here, we discuss the spatial resolution for hypocenter determination, using the configuration of the fiber-optic cable at Azuma volcano. This cable runs primarily in the north–south direction and is winding in some places, especially in the southern part. We placed nine pseudo hypocenters at a depth of − 1 km around the fiber-optic cable. When the hypocenter is located close to the fiber-optic cable, the resolution becomes good for the arrival time difference method. On the other hand, when the hypocenter is located far from the fiber-optic cable, the errors are large far from the cable (Figure S6). The resolution is not good in the east–west direction for the ASL method (Figure S7). The arrival time differences can constrain the location roughly in the east–west direction and the amplitude in the north–south direction. In other words, we may determine the source locations for volcanic earthquakes and tremors by the combined use of these two methods, even when the fiber-optic cable does not surround their source locations.

### Measurement of arrival time difference

Since the fiber-optic cable covers a long distance, we have many choices for the interval distance of a pair of measurement points, for which the arrival time difference is calculated. We set the maximum interval distance to be approximately one quarter the wavelength of the waves to stably measure the arrival time differences from correlated waveforms. We analyzed the waveforms for a pair of measurement points located within a distance of 90 m along the cable, which is approximately one fourth of the wavelength at 4 Hz of the S-wave. In order to examine waveform correlations, we used waveforms with a coherence of greater than 0.5. Since the dynamic strain signals at measurement points with different cable directions may show different polarity, we used only the pairs of measurement points for which the cable directions were matched with each other within less than 30°.

We calculated nine arrival time differences for a pair of measurement points within 91.8 m along the cable. Then, we determine which intervals were suitable, applying the following procedure. (1) We prepared data for nine intervals from 10.2 to 91.8 m in steps of 10.2 m. (2) We obtained the best location and evaluated the variance reduction for each interval. (3) We removed data for intervals for which the variance reduction was less than 10%. (4) The process was then repeated from step (2). The processes from (2) to (4) are terminated when the variance reduction was greater than 10%. We analyzed the volcanic earthquake on July 4, and determined the intervals of 30–70 m for the measurement of arrival time difference for all the volcanic earthquakes we analyzed.

### Estimation of site amplification factor

Coda waves, which follow the direct S-waves, are uniformly spatially distributed when the heterogeneous structure sufficiently scatters the seismic waves^36^. The site amplification factors were estimated by measuring the relative amplitude of the coda wave at target sites with respect to the reference station^37^. Since large earthquakes accompany coda waves long enough for the analyses, we considered 11 tectonic earthquakes with magnitudes of larger than or equal to 3 that occur within a distance of approximately 50–200 km and a depth of approximately 10–100 km. Figure S8 shows the epicentral maps and Figure S9 represents seismic records of these earthquakes at MNP800. We calculated the root mean square amplitudes at the measurement points along the cable, and compared them to that at the reference point (MP1027 where a seismometer was temporally placed). We generally used coda waves with a lapse time of larger than twice the direct S-wave travel time^36^ in order to reduce the effects of the radiation pattern of S-waves. Since the tectonic earthquakes we analyzed are located far from Azuma volcano, the effects of the radiation pattern were very small. We estimated the relative amplitudes for lapse times of 0–50 s every 5 s and the averaged amplitude at each measurement point is used as site amplification factors in our analyses.

We calculate the relative amplitudes for different lapse times (approximately 0–50 s) obtained from the arrival times of S-waves at 2–6 Hz. The standard deviations of the relative amplitudes are quite small, which shows that the site factors are reliably evaluated (Figure S10). The site amplification factor changes along the fiber-optic cable with an order of approximately 1 on the logarithmic scale. This suggests that this correction is quite important for hypocenter determination using the ASL method.

### Numerical simulation of seismic wave propagation

We numerically calculated seismic waves from a source in order to evaluate the effect of volcano topography on the seismic wave amplitude using the finite difference method^38^. Dynamic strains of 2–6 Hz are calculated at the measurement points along the fiber optic cable. We set the topography of Azuma volcano using a 5-m digital elevation model in a calculation region of 15 km × 15 km × 5 km. The grid size was set to 5 m. The depth of the receivers was set to 20 m from the surface in order to avoid instability in the strain calculations.

For the simulation to examine the effect of topography in Subsurface structure obtained from analyses of regional tectonic earthquakes, we used a velocity structure with a constant P-wave velocity of 5.1 km/s and S-wave velocity of 3 km/s. S-wave is vertically incident from the bottom of calculation domain.

For calculating the amplitude distributions of seismic sources beneath Jododaira, we simplified the velocity structure (Figure S2) into four layers. Velocity boundaries were parallel to the topography, and their depths were 1.0, 2.1, and 3.1 km from the free surface. Random velocity fluctuations were superimposed on the top layer because some previous studies showed strong short-wavelength heterogeneity in shallow regions^29,30^. An exponential autocorrelation function with a characteristic scale of 0.1 km and a root mean square fractional fluctuation of 0.1 was used to represent the heterogeneity. The hypocenter was set at a depth of − 1 km beneath Jododaira. Three types of source mechanism were assumed: (1) isotropic source, (2) DC1: a double-couple source with a strike of 70°, a dip of 70°, and a rake of 0°, and (3) DC2: a double-couple source with a strike of 0°, a dip of 90°, and a rake of 0°.

## Supplementary Information

Supplementary Figures.

## Data Availability

Due to the very large size of the fiber-optic data, the decimated digital data are available upon request.
