# Supplementary material for: Source location of volcanic earthquakes and subsurface characterization using fiber-optic cable and distributed acoustic sensing system
Source: Sci Rep. 2021 Mar 18;11:6319. doi: 10.1038/s41598-021-85621-8 (PMC7973575; doi:10.1038/s41598-021-85621-8)
Supplement: Supplementary file 1 — Supplementary Figures. [file 41598_2021_85621_MOESM1_ESM.docx]

**Supplementary Figures**

Source location of volcanic earthquakes and subsurface characterization using fiber-optic cable and distributed acoustic sensing system

Takeshi Nishimura^1^, Kentaro Emoto^1^, Hisashi Nakahara^1^, Satoshi Miura^2^, Mare Yamamoto^2^, Shunsuke, Sugimura^2^, Ayumu Ishikawa^1^, Tsunehisa Kimura^3^

**
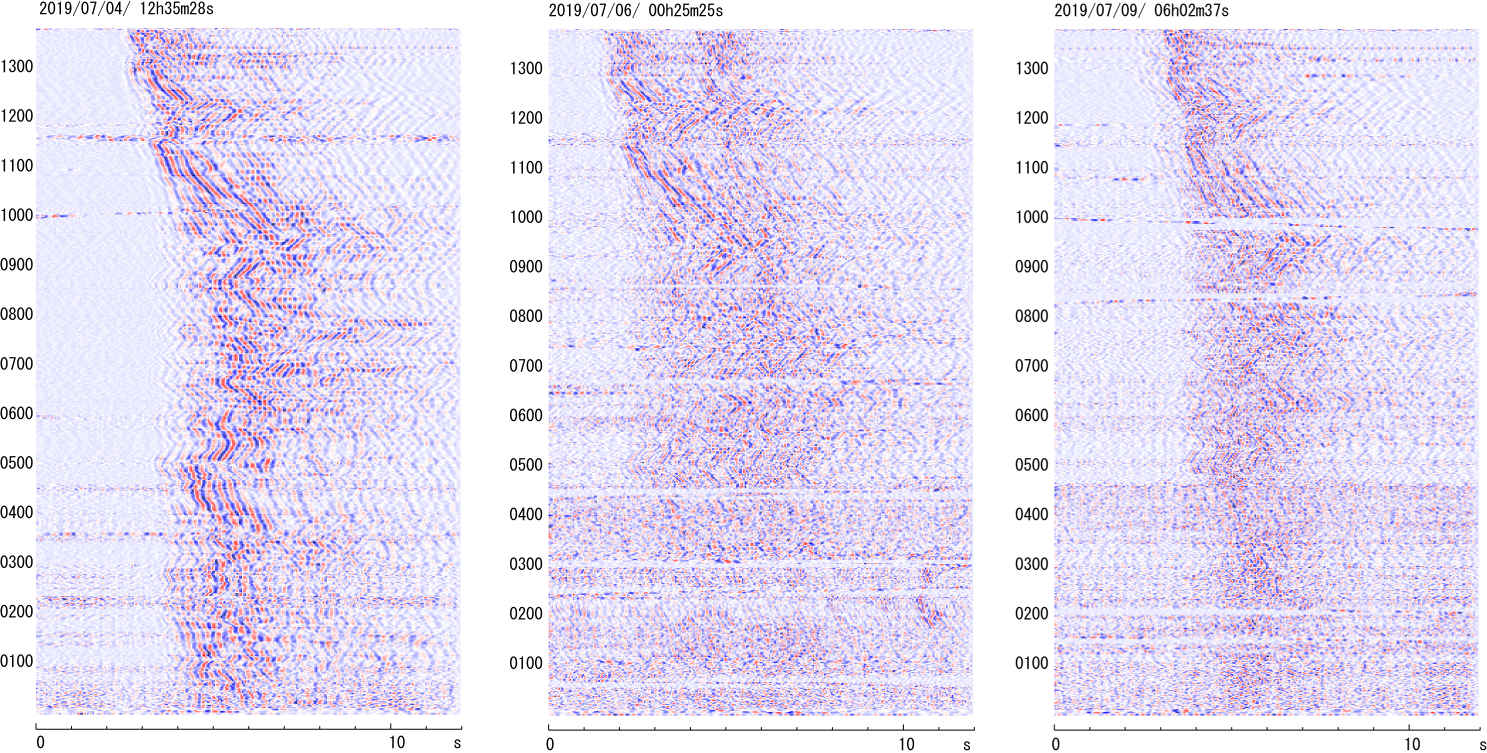
**

Figure S1(a). Record sections of volcanic earthquakes on July 4, July 6 and July 9. The seismograms are filtered at 2-6 Hz.

**
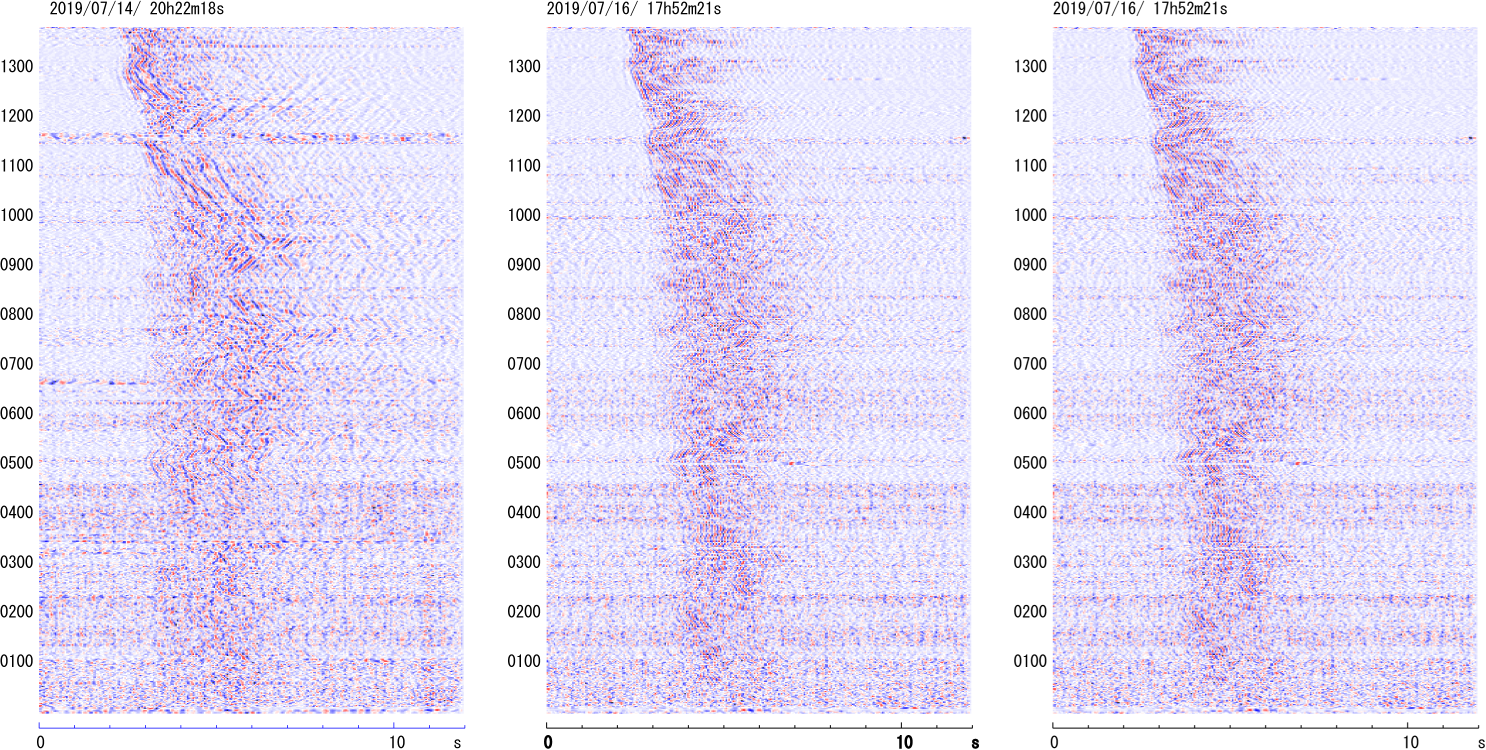
**

Figure S1(b). Record sections of volcanic earthquakes on July 14 and two on July 16. The seismograms are filtered at 2-6 Hz.

**
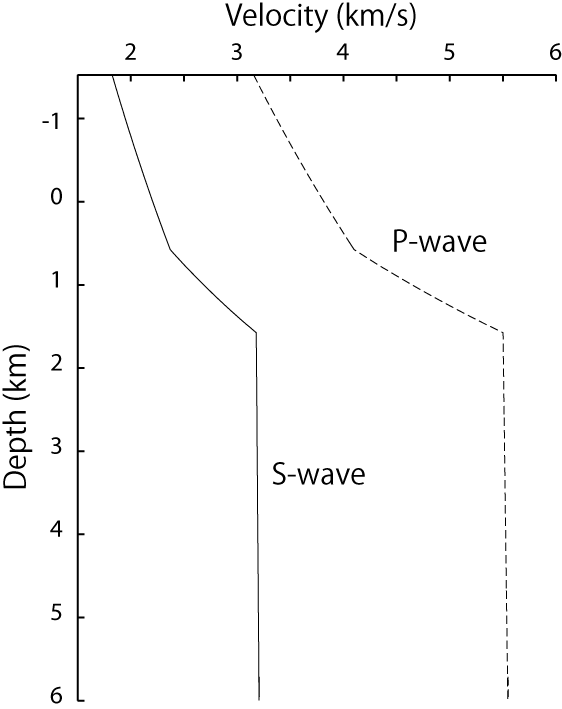
**

Figure S2. S-wave and P-wave velocity structures used for hypocenter determination. The P- to S-wave velocity ratio is 1.73.

**
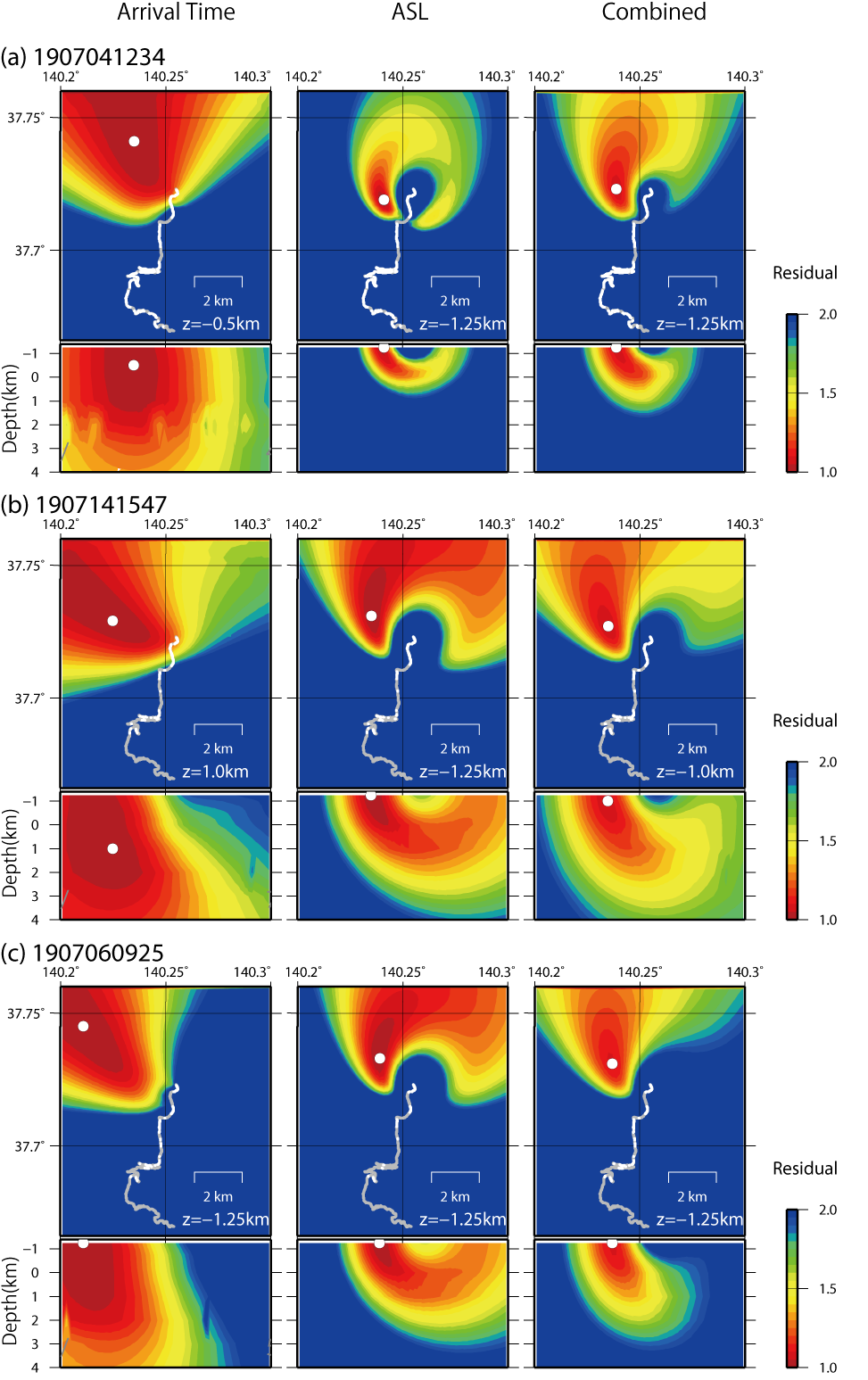
**


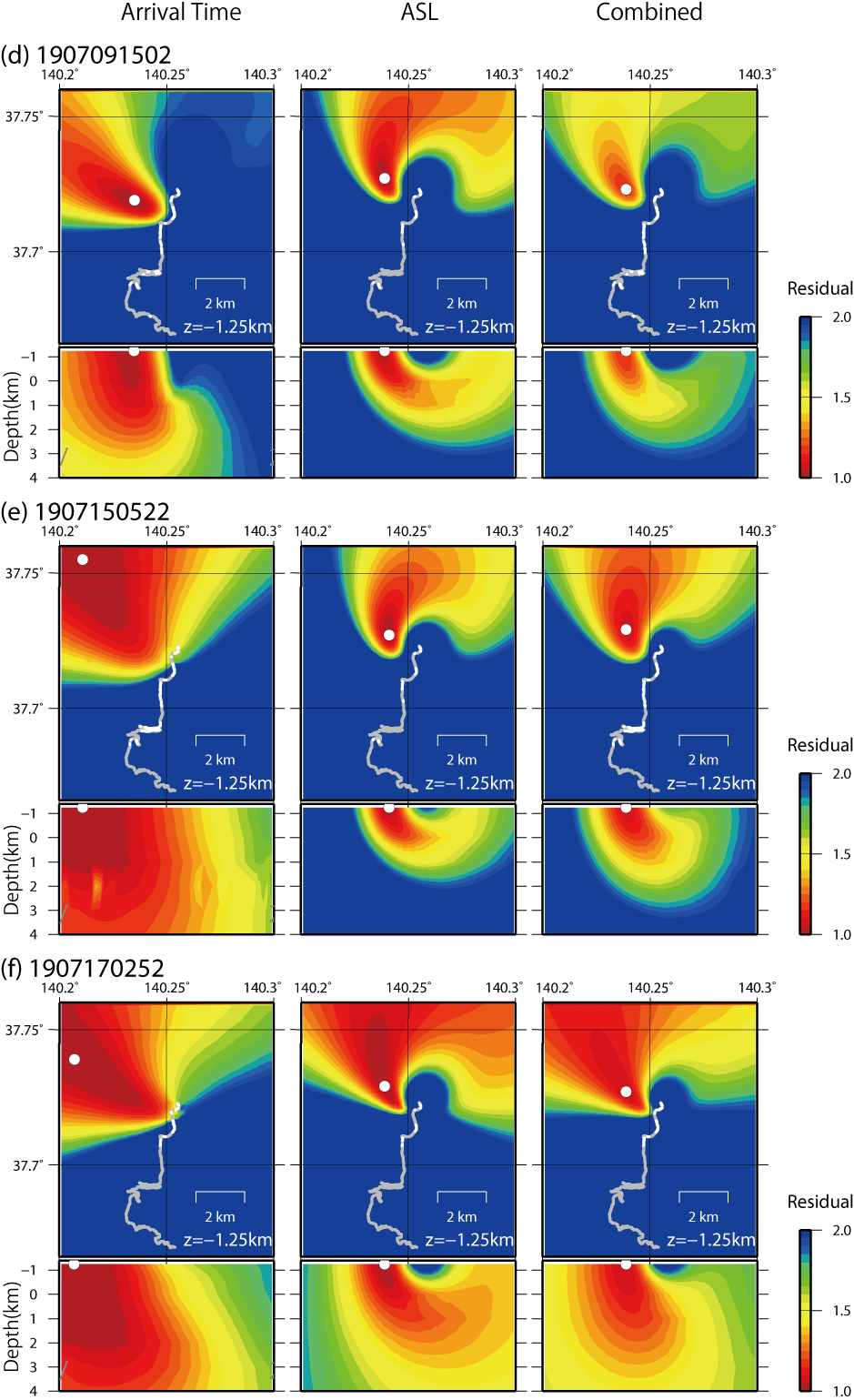


Figure S3. Source locations of the volcanic earthquakes and spatial distribution of the residual between the observed and theoretical values for each of the six volcanic earthquake. The left, middle, and right side panels represent the results for the arrival time difference method, ASL, and combined method, respectively. White lines indicate the locations of the fiber-optic cable where the data are used for each of the determination method while the grays are the locations where the data are not used. The residuals in (a) and (b) are normalized by the minimum residual for each method. The residual in (c) is *S* in Eq. (2). This figure was created by Generic Mapping Tools (GMT) v4.5.5^39^.


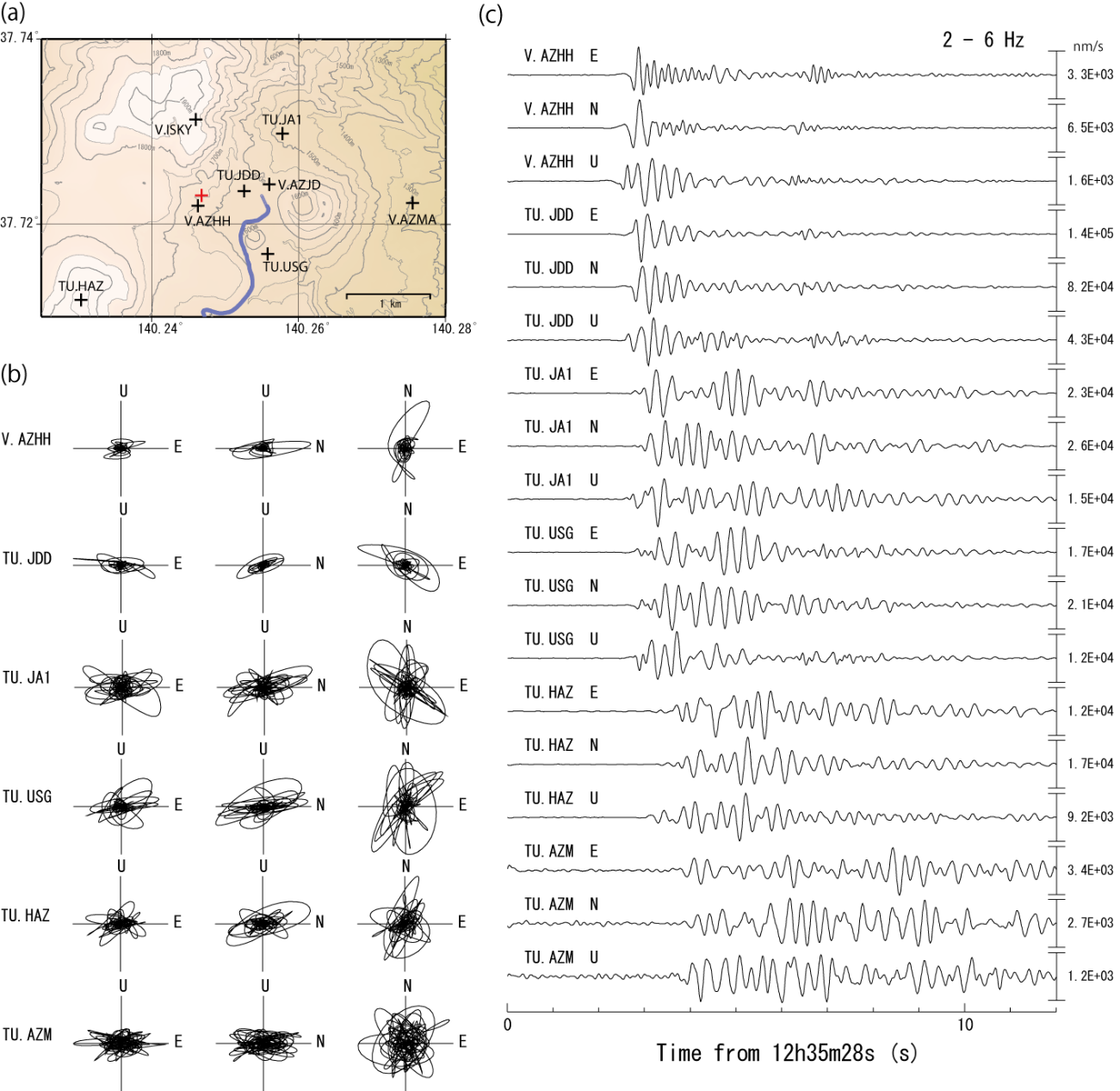


Figure S4. Particle orbits for the volcanic earthquake on July 4, 2019. (a) Locations of permanent stations (black pluses), hypocenter of the volcanic earthquake (red plus), and fiber-optic cable (purple line). (b) Particle orbits for permanent stations. Here, U, E, and N represent the up, east, and north directions. Several stations (V.AZJD and V.ISKY) are not shown because of noise contamination. (c) Three component velocity seismograms. The waveforms are filtered at 2-6 Hz. Figure (a) was created by Generic Mapping Tools (GMT) v4.5.5^39^.


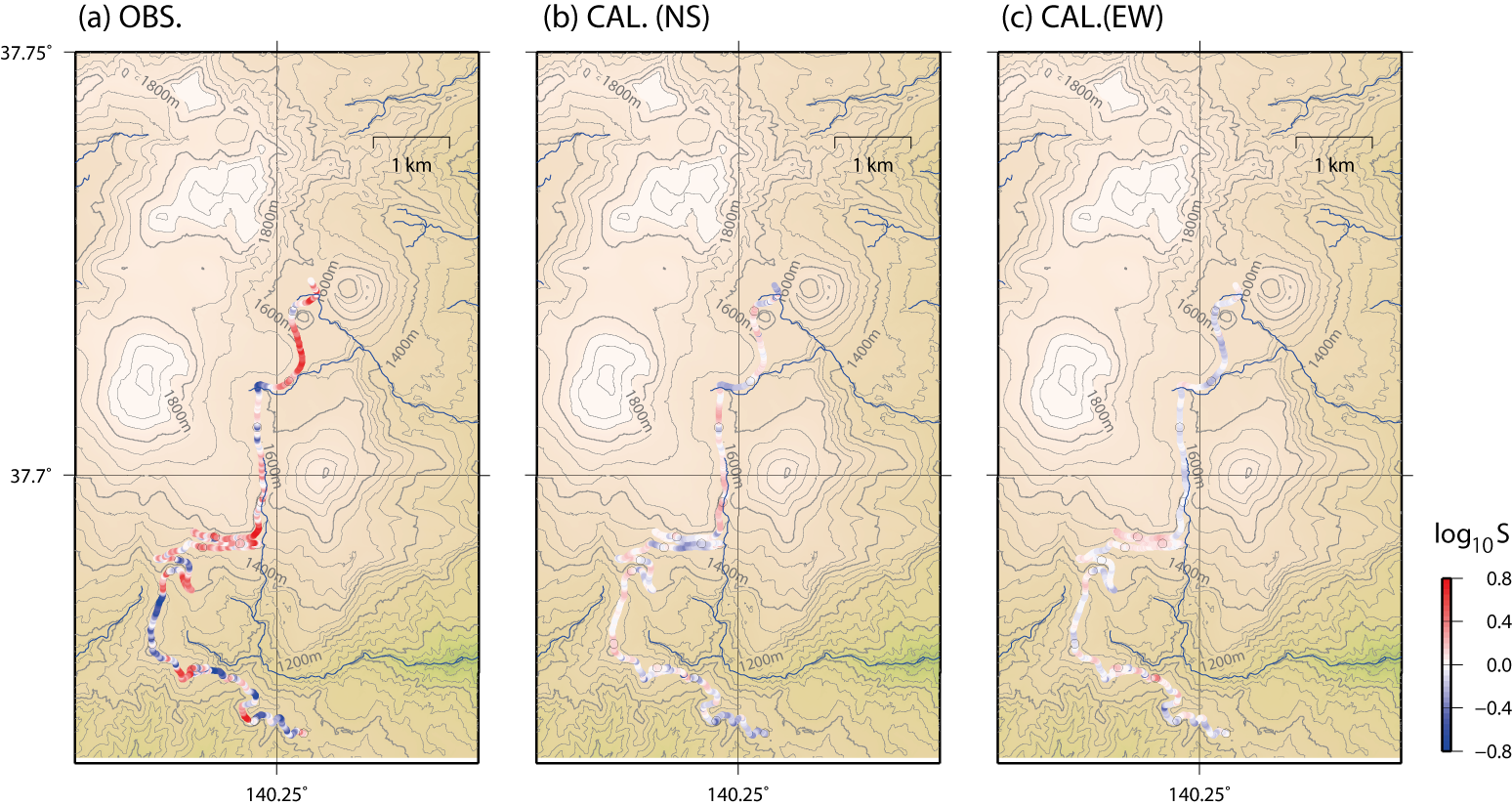
Figure S5. Site amplification factors along the fiber-optic cable. (a) Estimated factors same to Figure 6. Site amplification factors estimated from the numerical simulation with a vertical incidence of S-wave oscillating (b) in the NS direction and (c) in the EW direction. This figure was created by Generic Mapping Tools (GMT) v4.5.5^39^.


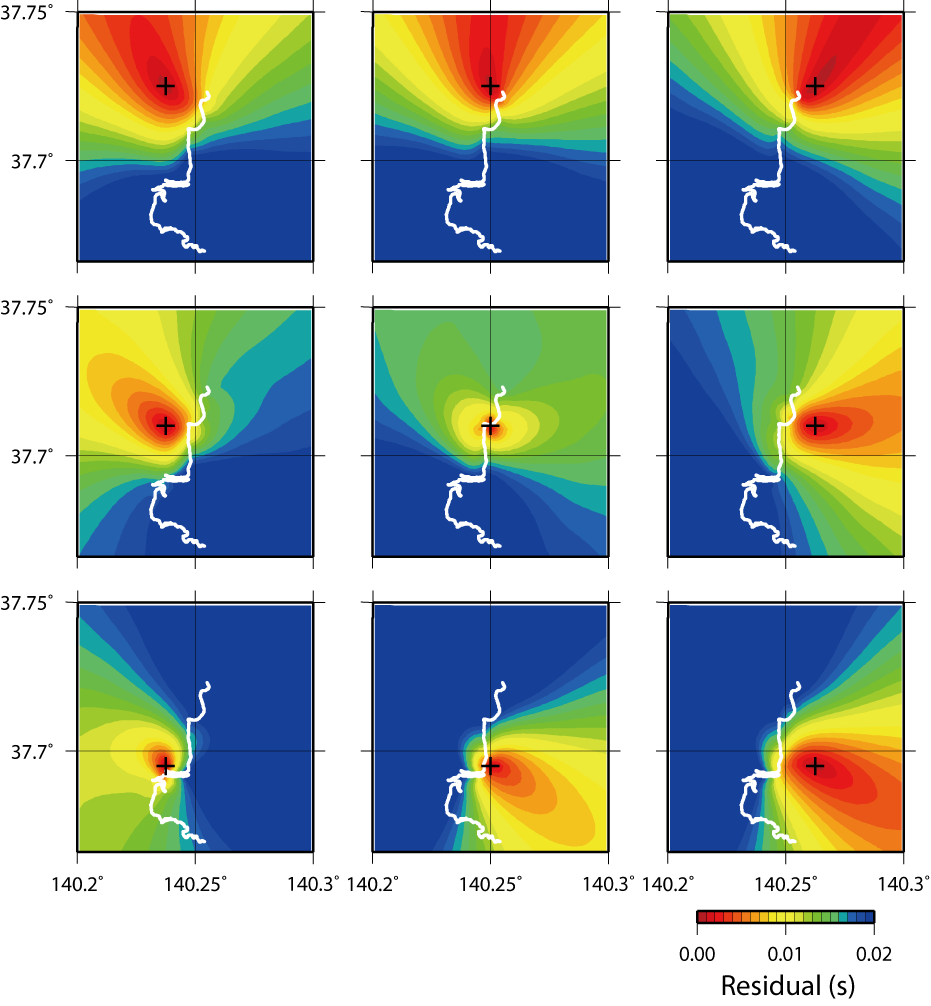


Figure S6. Spatial distribution of residuals between the simulated arrival time differences and the predicted arrival time differences for nine pseudo hypocenters. The simulated arrival time differences are calculated at all measurement points with a channel number greater than 500 along the fiber cable for an assumed seismic source location (plus symbols). The residuals, which are the root mean squared differences averaged for all measurement points with a channel number larger than 500 along the fiber-optic cable, are represented by a color contour every 0.02º × 0.02º in the NS and EW directions. This figure was created by Generic Mapping Tools (GMT) v4.5.5^39^.


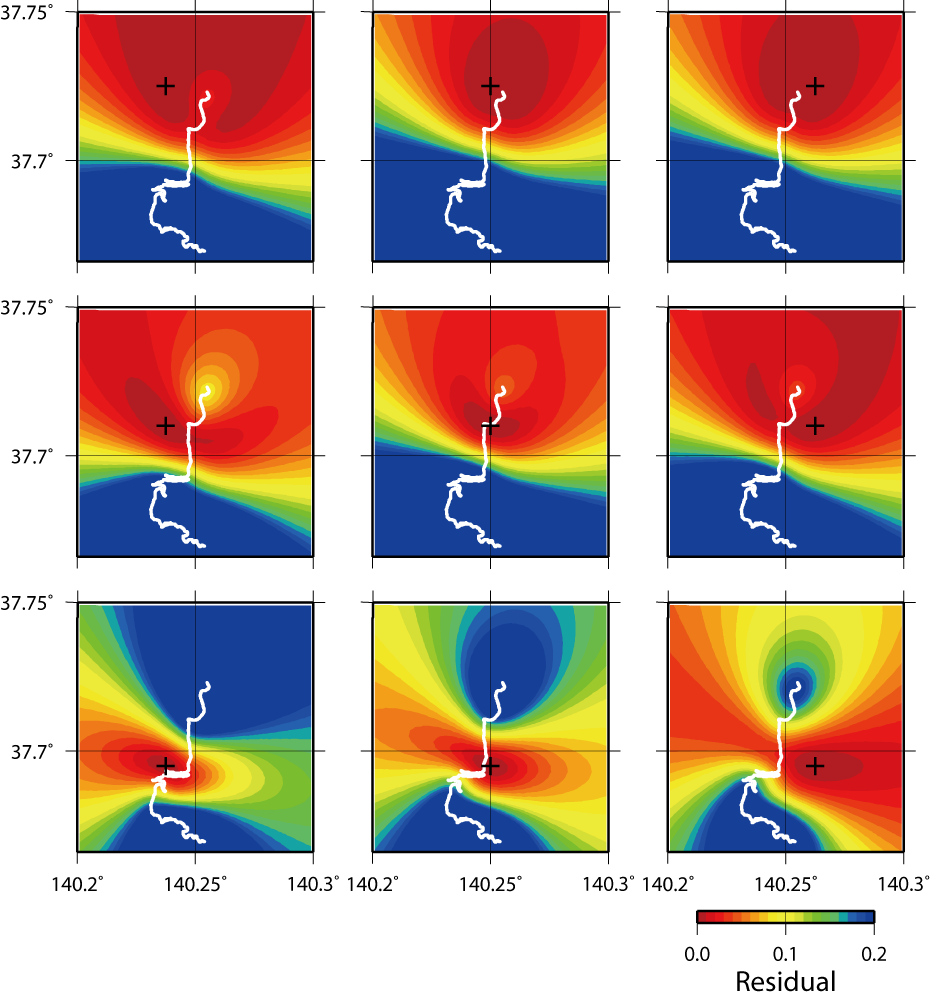


Figure S7. Spatial distribution of residuals between the simulated seismic amplitudes and the predicted seismic amplitudes for nine pseudo hypocenters. The simulated seismic amplitudes are calculated at all measurement points with a channel number greater than 500 along the fiber cable for an assumed seismic source location (plus symbols). The residuals, which are the root mean squared differences averaged for all measurement points with a channel number larger than 500 along the fiber-optic cable, are represented by a color contour every 0.02º × 0.02º in the NS and EW directions. This figure was created by Generic Mapping Tools (GMT) v4.5.5^39^.

**
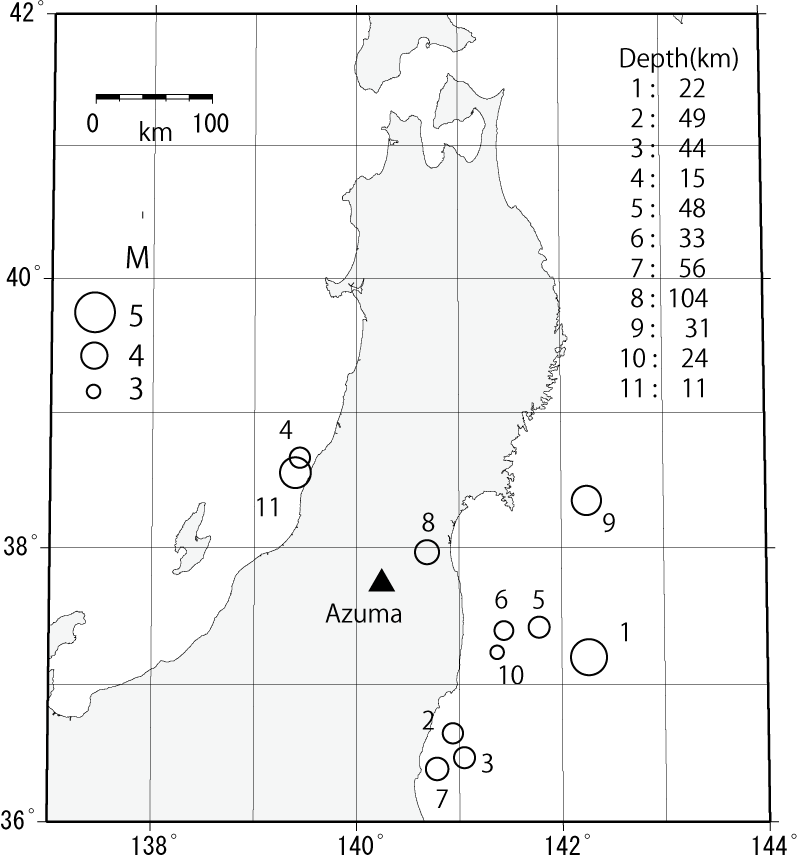
**

Figure S8. Epicenters of regional tectonic earthquakes used for estimating the site amplification factors. Epicenters are denoted by open circles, the radius of which represents the magnitude of the earthquake. The numbers represent the earthquakes whose waveforms are shown in Figure S9. This figure was created by Generic Mapping Tools (GMT) v4.5.5^39^.


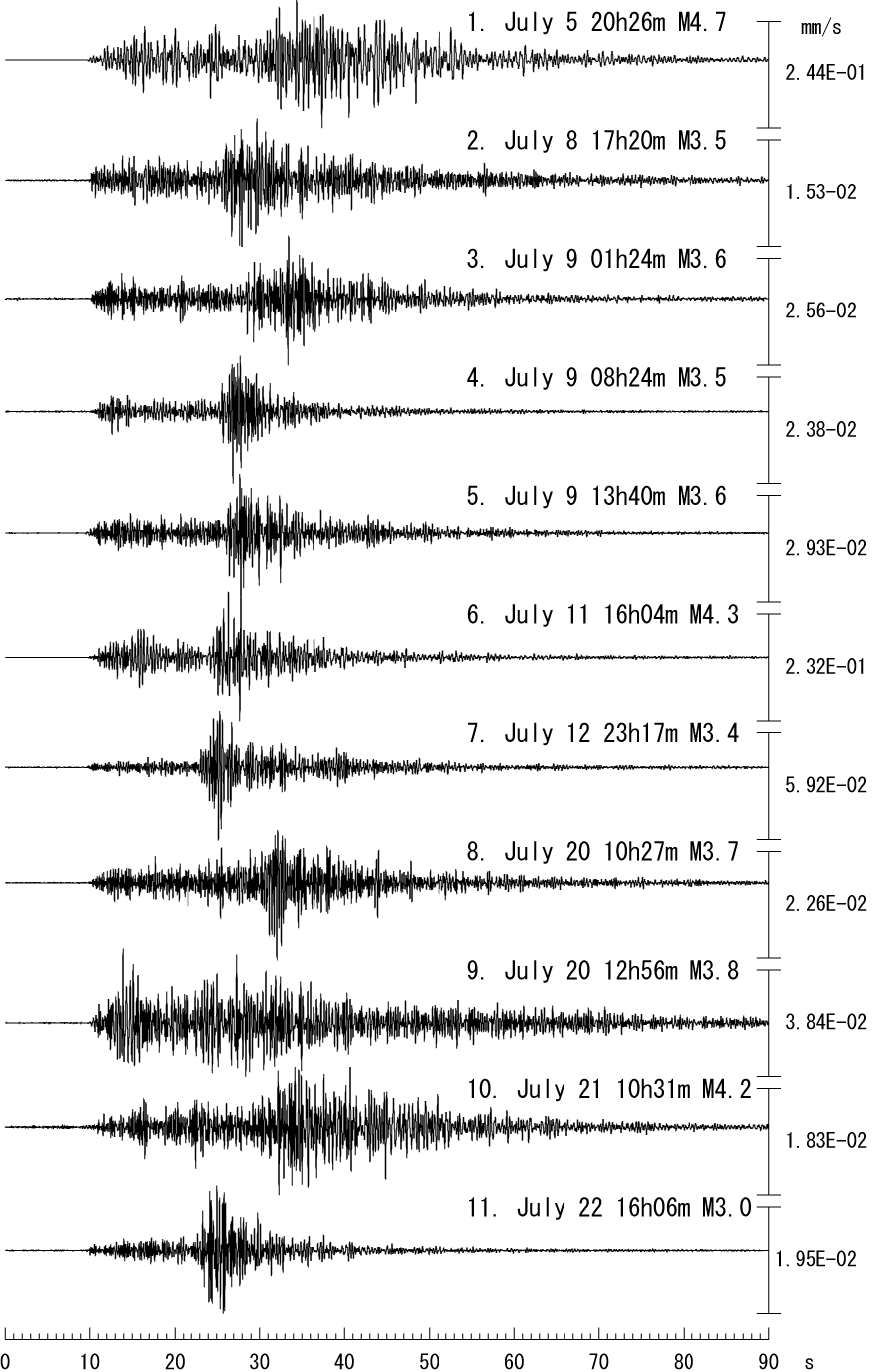


Figure S9. Seismic waves of the 11 regional tectonic earthquakes recorded at MNP800 by the DAS system. The hypocenters are shown in Figure S8.


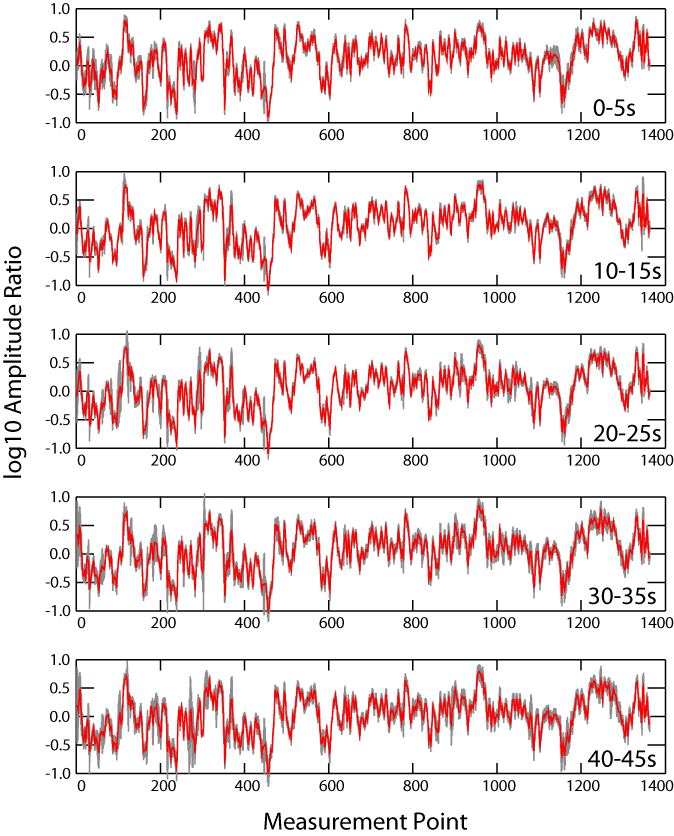


Figure S10. Site amplification factors determined from coda waves at different lapse times from S-wave onsets. The red lines represent the median values of site amplification factors for the 11 earthquakes shown in Figure S8 and gray bards indicate one standard deviation at each measurement point.
